# Spatial stress profiling by picosecond ultrasonics with X-rays using buried detection layers

**DOI:** 10.1016/j.pacs.2026.100871

**Published:** 2026-08-17

**Authors:** M. Mattern, F.-C. Weber, M. Rössle, J.-E. Pudell, A. Jurgilaitis, B. Ahn, F. Baltrusch, J.C. Ekström, M. Herzog, J. Jarecki, D. Kroon, L. Mehner, C. Walz, S.P. Zeuschner, J. Larsson, M. Kronseder, M. Bargheer, A. von Reppert, D. Schick

**Affiliations:** a Max-Born-Institute for Nonlinear Optics and Short Pulse Spectroscopy, 12489 Berlin, Germany; b Institut für Physik und Astronomie, Universität Potsdam, 14476 Potsdam, Germany; c Helmholtz-Zentrum Berlin für Materialien und Energie GmbH, Wilhelm-Conrad-Röntgen Campus, BESSY II, 12489 Berlin, Germany; d European X-ray Free-Electron Laser Facility, 22869 Schenefeld, Germany; e MAX IV Laboratory, Lund University, PO Box 118, 221 00 Lund, Sweden; f Department of Physics, Lund University, PO Box 118, 221 00 Lund, Sweden; g LINXS Institute of advanced Neutron and X-ray Science, Mesongatan 4, 224 84 Lund, Sweden; h Institute of Experimental and Applied Physics, University of Regensburg, 93053 Regensburg, Germany

**Keywords:** Picosecond ultrasonics, Stress profiling, Ultrafast X-ray diffraction, Heterostructures

## Abstract

Understanding the dynamics of laser-excited nanolayers thicker than their optical absorption length requires access to the inhomogeneous energy distribution along the depth. Here, we demonstrate the quantitative extraction of the excitation profile from the launched picosecond strain pulse detected in a buried crystalline layer using ultrafast hard-X-ray diffraction. We apply this approach to Ni and Cu thin films, revealing stress profiles that differ from the optical absorption profile because of rapid electron-mediated energy redistribution. We further examine the influence of the detection layer thickness and an insulating interlayer on the profiling sensitivity. Resolving the subtle differences in the excitation profile in Ni for 400 and 800 nm pump pulses highlights the power of our approach and benchmarks the experimental sensitivity. With a crystalline buried detection layer as the only requirement, our approach is broadly applicable to quantitatively study the spatial energy distribution even in amorphous layers of functional materials.

## 1 Introduction

In time-resolved pump-probe experiments, the timescale of evolving material properties encodes non-equilibrium microscopic processes [1], [2], [3], [4], [5], [6], [7], [8], which open new perspectives on material design and control for future applications. However, in layers with thicknesses exceeding their optical penetration depth, the inhomogeneous deposition of energy can influence the observed dynamics averaged over the probed volume [9], [10], [11], [12], especially if the laser-induced response is nonlinear in nature [7], [8], [13], [14], [15], [16]. Thus, identifying the intrinsic physical mechanisms and disentangling material-related and externally introduced heterogeneities [9], [10], [16], [17] requires experimental access to this spatial excitation profile.

In general, rapidly depositing energy into a material induces a stress on the lattice driving picosecond lattice dynamics, e.g. launching strain pulses propagating through the sample structure [18], [19], [20], [21], [22]. The initial shape of this strain pulse is dictated by the spatial stress profile that is the superposition of contributions from the different degrees of freedom. Picosecond ultrasonics employs this relation to access the spatial stress profile by probing the shape of the strain pulse [18], [19], [20]. In all-optical experiments, the shape of the strain pulse is accessed either by its phonon spectrum [23], [24] or characteristic reflectivity changes when the strain pulse enters and leaves the probed volume at the near-surface region defined by the optical penetration depth [18], [25], [26].

Ultrafast hard-X-ray diffraction (UXRD) experiments directly probe the effect of the strain pulse on the lattice via the shift [27], [28] and the changing shape [29], [30] of the layer‘s Bragg peak along the out-of-plane direction in reciprocal space. The latter encodes the laser-induced heterogeneity of the interatomic distance throughout the layer. Modeling the transient shape of the Bragg peak allows quantification of this heterogeneity originating from an inhomogeneous excitation or traversing strain pulses [30], [31]. This approach works well for high-quality crystalline layers [30], [31]. However, it becomes less reliable for inhomogeneous polycrystalline layers and materials exhibiting additional mechanisms changing the shape of the Bragg peaks such as phase transitions. In contrast, the shift of Bragg peaks encoding the change of the average lattice constant can be reliably accessed even in polycrystalline or mosaic layers employing X-ray sources of limited monochromaticity and brilliance [20], [32]. In general, the transient average lattice constant is determined by a quasi-static expansion from heating the layer and characteristic signatures of the propagating strain pulses [20]. The delay of these signatures was shown to provide insights into the nanomorphology of layers [33], [34] and even transient spatial phase heterogeneities [35], [36]. On the other hand, the shape of these signatures in the strain response of a buried detection layer was shown to enable access to the spatial excitation profile of different degrees of freedom [8], [21], [37], [38].

Here, we further develop this experimental approach to spatial stress profiling via picosecond ultrasonics with X-rays (PUX) using buried detection layers. We exemplify the stress profiling in systematically varied Ni-Au and Cu-Au heterostructures and derive guidelines for optimizing the sensitivity of spatial stress profiling through the sample design. In Section 2, we illustrate the conceptual idea of accessing the excitation profile via PUX experiments on heterostructures consisting of a photo-excited layer of interest and a buried detection layer. The comparison with experiments on Cu-Au and Ni-Au heterostructures identifies the rapid, pronounced heating of the detection layer as the main challenge for recording isolated signatures of the strain pulse launched by the layer of interest. In Section 3, we exemplify the extraction of the spatial stress profile for Cu and Ni. Section 4 showcases the influence of the heterostructure design on the sensitivity of the spatial stress profiling and benchmarks the potential sensitivity by resolving the subtle difference in the spatial stress profile of Ni upon 400 and 800nm excitation. Based on these results, we develop a guideline for spatial stress profiling via PUX by discussing the selection of the optimal detection layer material and thickness as well as strategies to overcome potential experimental challenges in Section 5.

**Fig. 1:**
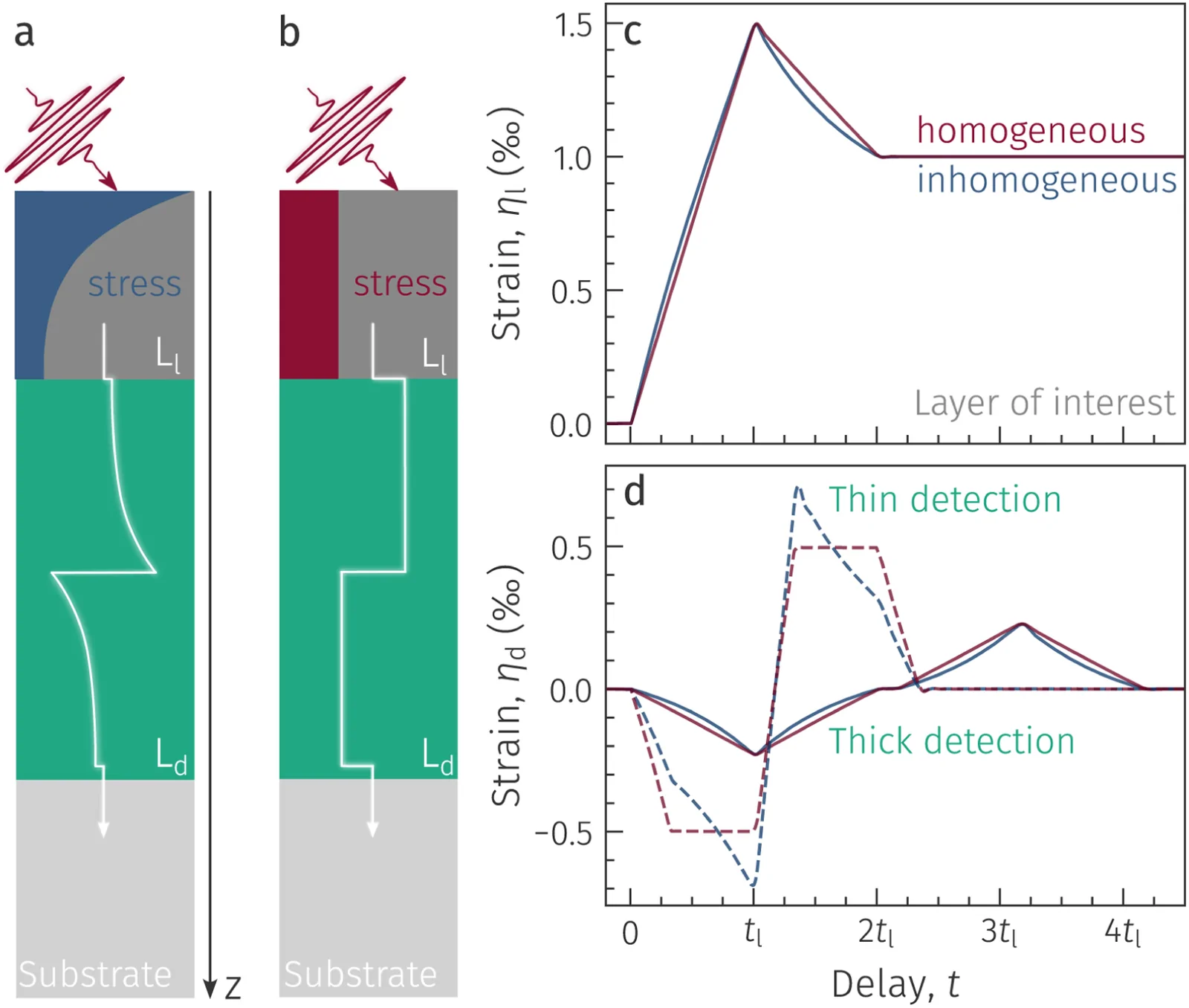
**Concept of stress profiling by PUX:** (a,b) Sketch of a sample structure consisting of the photo-excited layer of interest (dark gray) and a buried detection layer (green). The laser excitation induces a stress on the lattice (red and blue shaded areas) whose spatial profile determines the shape of the strain pulse (white solid line) propagating through the sample. (c) The corresponding strain response of the layer of interest for the homogeneous (red) and inhomogeneous stress profile (blue). (d) The same for the buried detection layer that translates the spatial shape of the strain pulse to the time domain via the speed of sound. The solid lines denote the strain response of a thick detection layer as sketched in (a) and (b). The dashed lines denote the strain response of a detection layer thinner than the spatial width of the strain pulse. In both cases the strain response depends on the shape of the stress profile and in the case of the thin detection layer even the amplitude changes with the stress profile.

## 2 PUX experiment

Probing the average strain of a buried detection layer translates the spatial shape of the launched strain pulse to the time domain. This fundamental idea of stress profiling by PUX using buried detection layers is illustrated in Fig. 1 for a perfectly impedance-matched heterostructure with a uniform sound velocity $v_{\text{s}}$ and density. The optical excitation of the layer of interest (dark gray) of thickness $L_{\text{l}}$ induces a stress on the lattice (red and blue shaded areas). Rapid energy redistribution along the sample depth [33], [39] or among the different degrees of freedom [21], [37] modifies its spatial shape with respect to the absorption profile. The stress launches a bipolar strain pulse (white solid line) whose spatial shape resembles that of the stress profile (c.f. Fig. 1a and b). The expansive part of the strain pulse reaching the detection-layer-interface at $t_{\text{l}}=L_{\text{l}}/v_{\text{s}}$ marks the delay of maximum expansion of the photo-excited layer and maximum compression of the buried detection layer that contains the leading compressive part. Subsequently, the expansive part propagates into the buried detection layer compensating its initial compression. At later delays, the propagation of the compression into the substrate results in a maximum expansion of the detection layer at $t_{\text{l}}+L_{\text{d}}/v_{\text{s}}$. After $2t_{\text{l}}+L_{\text{d}}/v_{\text{s}}$ the strain pulse has propagated completely into the substrate and the strain of the detection layer vanishes. These signatures of the traversing strain pulse in the layer-specific strain response uniquely depend on the shape of the strain pulse (c.f. Fig. 1c and d) . This enables the extraction of the spatial stress profile from the transient strain response recorded in PUX experiments by tracking the shift of the Bragg peaks in reciprocal space. If the detection layer is thinner than the dimension of the strain pulse, the shape of the strain pulse not only governs the temporal shape but also the amplitude of the signatures in the transient strain response.

**Fig. 2:**
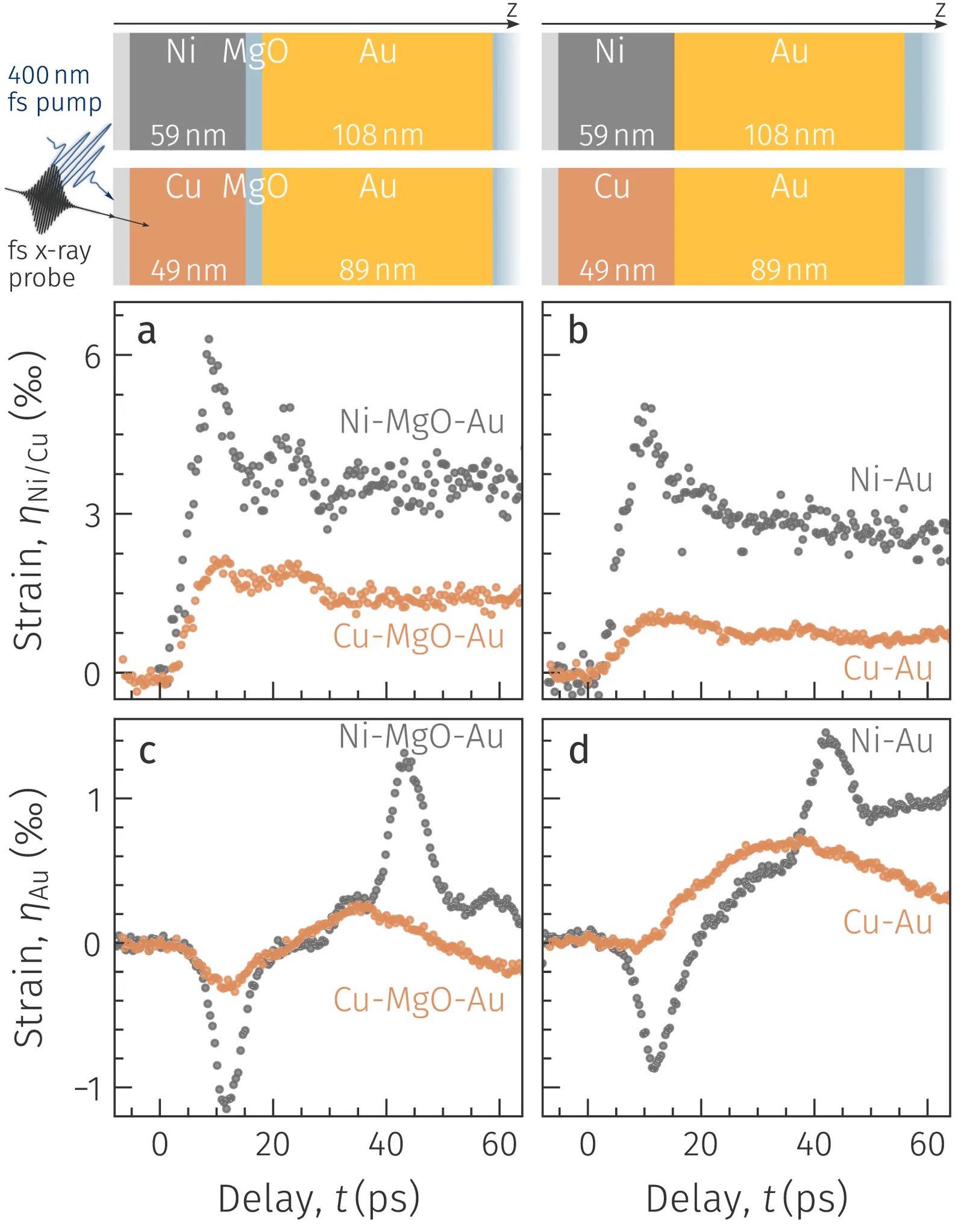
**PUX experiment on Ni-Au and Cu-Au heterostructures:** (a,b) Strain response of the Ni (gray) and Cu (orange) layers of the heterostructures with (a) and without an insulating MgO interlayer (b). (c,d) Corresponding strain response of the buried Au detection layers. In the presence of an MgO layer, the Au layers show no quasi-static expansion from heating. In the absence of an interlayer, the Au detection layers are heated either slowly or rapidly in the case of a Ni or Cu layer, respectively. In the case of the Cu-Au heterostructure, the rapid excitation of the Au layer even compensates its initial compression.

We conducted PUX experiments at the FemtoMAX beamline at the MAX IV Laboratory [40] and tracked the layer-specific lattice response upon excitation by a 50fs laser pulse with a central wavelength of 400nm via ultrafast symmetric X-ray diffraction employing 100fs hard X-ray pulses with a photon energy of 8keV. Further details on the experiment and the sample growth are provided in the Methods section. Fig. 2 displays the layer-specific strain response of four heterostructures consisting of photo-excited Ni or Cu layers on top of Au detection layers that are approximately twice as thick with and without an insulating 7.5nm-thick MgO interlayer. The samples are capped by 7.5nm of aluminum oxide to prevent oxidation.

Comparing the strain response of the Ni and Cu layers (Figs. 2a and b) and the strain response of the Au detection layer (Figs. 2c and d) with the idealized situation in Figs. 1c and d reveals two major differences in real experiments: (i) The acoustic impedance mismatch between the layers results in additional signatures of the propagating strain pulse in the layer-specific strain response due to partial reflections at interfaces. (ii) In metallic heterostructures without an insulating interlayer the rapid energy transport into the buried detection layer and the associated thermal expansion impedes background-free detection of the signatures of the launched strain pulse. In the case of the Cu-Au heterostructure (see Fig. 2d) the strain response of the buried Au layer is even dominated by its rapidly rising intrinsic expansion and no pronounced initial compression is observed. Thus, extracting the signatures of the launched picosecond strain pulse would require modeling the energy transport into the Au layer as well as its expansion which drastically increases the number of parameters and accordingly the uncertainty of the extracted shape of the strain pulse. This means that stress profiling via PUX experiments using buried detection layers generally benefits from minimizing rapid excitations of the buried detection layer.

**Fig. 3:**
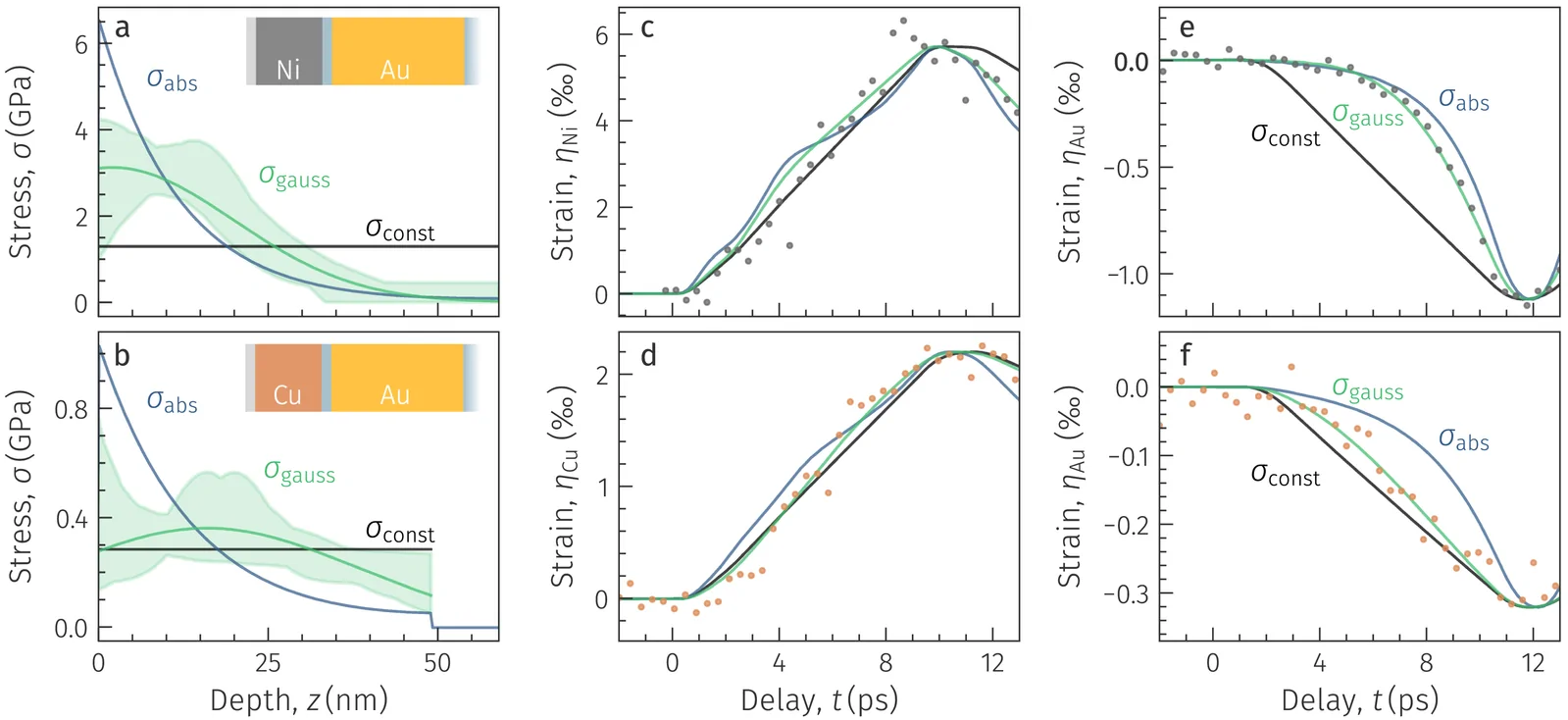
**Extraction of the spatial stress profile:** (a,b) Comparison of the extracted Gaussian-shaped stress profiles in Ni and Cu for heterostructures with MgO interlayers (green solid lines) with stress profiles proportional to the calculated optical absorption profiles (blue solid lines) and homogeneous stress profiles (black solid lines). The shaded area denotes the corresponding 2σ confidence interval. (c,d) Strain response of the Ni and Cu layers, respectively, (symbols) compared with the modeled strain response (solid lines) for the stress profiles in (a,b). (e,f) Same for the buried Au detection layer within the respective heterostructure. The pronounced deviations of the simulations based on $\sigma_{z,\text{abs}}$ and $\sigma_{z,\text{const}}$ from the measured Au response illustrate that the strain response of the buried detection layer is sensitive to the spatial stress profile. In contrast, the strain response of the photo-excited Ni or Cu layer is only weakly affected by the assumed stress profile. While electronic energy transport results in a rather homogeneous stress profile in Cu, the stress profile in Ni remains inhomogeneous.

## 3 Spatial stress profiling

In the following, we exemplify the extraction of the spatial stress profile in Ni and Cu employing the strain response of the buried, thick Au detection layers in the presence of insulating MgO interlayers that ensure a background-free detection of the strain pulse (see Fig. 2c).

In the framework of our forward approach, we start by assuming a spatio-temporal stress σ(z,t) within the photo-excited layer that drives the spatio-temporal strain response η(z,t) according to the linear elastic wave equation [20]. Dynamical X-ray scattering calculations relate η(z,t) to a transient shift of the layer-specific Bragg peaks that yields the strain response of the Ni, Cu and Au layers $\eta_{\text{Ni/Cu/Au}}\left(t\right)$, which we compare to the experiment. We conduct these calculations in the framework of the modular python toolbox udkm1Dsim [41]. By varying σ(z,t) and minimizing the quadratic deviation $\chi^{2}$

(1) $$\chi^{2}=\underset{t>0}{\sum }{\left(\eta_{d}^{\text{sim}}\left(t\right)-\eta_{d}^{\text{exp}}\left(t\right)\right)}^{2}$$

between the simulated $\eta_{d}^{\text{sim}}\left(t\right)$ and measured $\eta_{d}^{\text{exp}}\left(t\right)$ strain responses of the buried detection layer, we identify the stress profile within the photo-excited Ni and Cu layers. Importantly, the quantity extracted by this procedure is the laser-induced lattice stress profile – its conversion into an energy-density profile requires knowledge of the relevant Grüneisen parameters and stress-generating degrees of freedom.

We choose a simplified factorized description of the spatio-temporal stress $\sigma \left(z,t\right)=\sigma_{z}\left(z\right)\cdot \sigma_{t}\left(t\right)$. Assuming a time-independent $\sigma_{z}$ is often a suitable approximation: The spatial stress profile in metals is typically determined by the redistribution of the absorbed energy via electrons [39], [42]. This is much faster than the strain formation that is given by the characteristic length scale of the stress profile or the thickness of the photo-excited layer divided by the speed of sound [20]. The subsequent coupling of electrons and phonons – typically representing the most relevant degrees of freedom in metals – governs the temporal stress profile:

(2) $$\sigma_{t}\left(t\right)=H\left(t\right)\left(\Gamma_{\text{el}}\cdot e^{-t/\tau }+\Gamma_{\text{ph}}\left(1-e^{-t/\tau }\right)\right)$$

where H(t) is the Heaviside function, τ is the material-specific electron–phonon relaxation time and $\Gamma_{\text{el}}$ and $\Gamma_{\text{ph}}$ are the material-specific electronic and phononic Grüneisen parameters [43], [44], respectively.

In this work, we describe the spatial stress profile by a Gaussian, i.e. the fundamental solution of the diffusion equation that accounts for the rapid energy redistribution via electrons upon laser excitation:

(3) $$\sigma_{z,\text{gauss}}=\sigma_{0}\left(e^{-{\left(z-b\right)}^{2}/2a^{2}}+c\right)\;,$$

with the stress amplitude $\sigma_{0}$. The three free parameters a, b and c allow the corresponding strain response $\eta_{\text{d}}^{\text{sim}}\left(t\right)$ to be optimized to match the experiment. We want to emphasize, that Eq. (3) is only meant to exemplify the spatial stress profiling and that the following analysis works for any kind of spatial profile and could easily be extended to situations where different degrees of freedom exhibit significantly different spatial profiles [21]. As a reference, we compare the experimentally determined $\sigma_{z,\text{gauss}}$ with a stress profile following the absorption profile calculated by a multilayer transfer matrix algorithm

(4) $$\sigma_{z,\text{abs}}=\sigma_{0}\cdot \alpha_{\text{multilayer}}\left(z\right)\;,$$

and a fully homogeneous stress profile

(5) $$\sigma_{z,\text{const}}=\sigma_{0}\;.$$

Note, $\sigma_{0}$ is fixed to ensure the same integral as the extracted $\sigma_{z,\text{gauss}}$. Accordingly, Eqs. (4), (5) only serve as limiting cases of vanishing and infinitely fast electronic energy transport within the photo-excited layer in order to illustrate the role of rapid electronic energy redistribution modifying the stress profile with respect to the absorption profile.

Fig. 3 displays our results for the Cu-Au and Ni-Au heterostructures with MgO interlayer. The green solid lines in Figs. 3a and b denote the extracted stress profiles for Ni and Cu, respectively. They correspond to the minimum quadratic deviation $\chi^{2}$ between the experiment and the corresponding modeled strain response $\eta_{\text{Au}}^{\text{sim}}$. For both Ni and Cu we observe a significant deviation from the absorption profile (blue solid line) beyond the 2σ confidence interval (green shaded area). This indicates a significant rapid spatial redistribution of the optically deposited energy via electrons. While in Cu this results in an approximately homogeneous stress profile (black solid line), the stress profile in Ni remains significantly inhomogeneous directly illustrating the influence of electron–phonon coupling strength on rapid electronic energy transport [39].

These differences for Ni and Cu are also reflected by the deviations among the Au strain corresponding to the extracted $\sigma_{z,\text{gauss}}$ and the limiting cases of Eqs. (4), (5) (see Figs. 3e and f). In contrast, $\eta_{\text{Ni/Cu}}^{\text{sim}}$ for (3)–(5) are barely distinguishable (see Figs. 3c and d). In fact, the variation in $\chi^{2}$ is ten times smaller than for the Au detection layers indicating a ten times smaller sensitivity of the transient average strain of the photo-excited layer to the excitation profile. This originates on the one hand from the dominant contribution of the rising expansion (see Fig. S1) and on the other hand from the strong influence of the aluminum-oxide capping layer whose imperfect description might lower the overall agreement between model and experiment. Consequently, employing buried detection layers for spatial stress profiling is not only more flexible and more broadly applicable but also yields a much larger sensitivity.

**Fig. 4:**
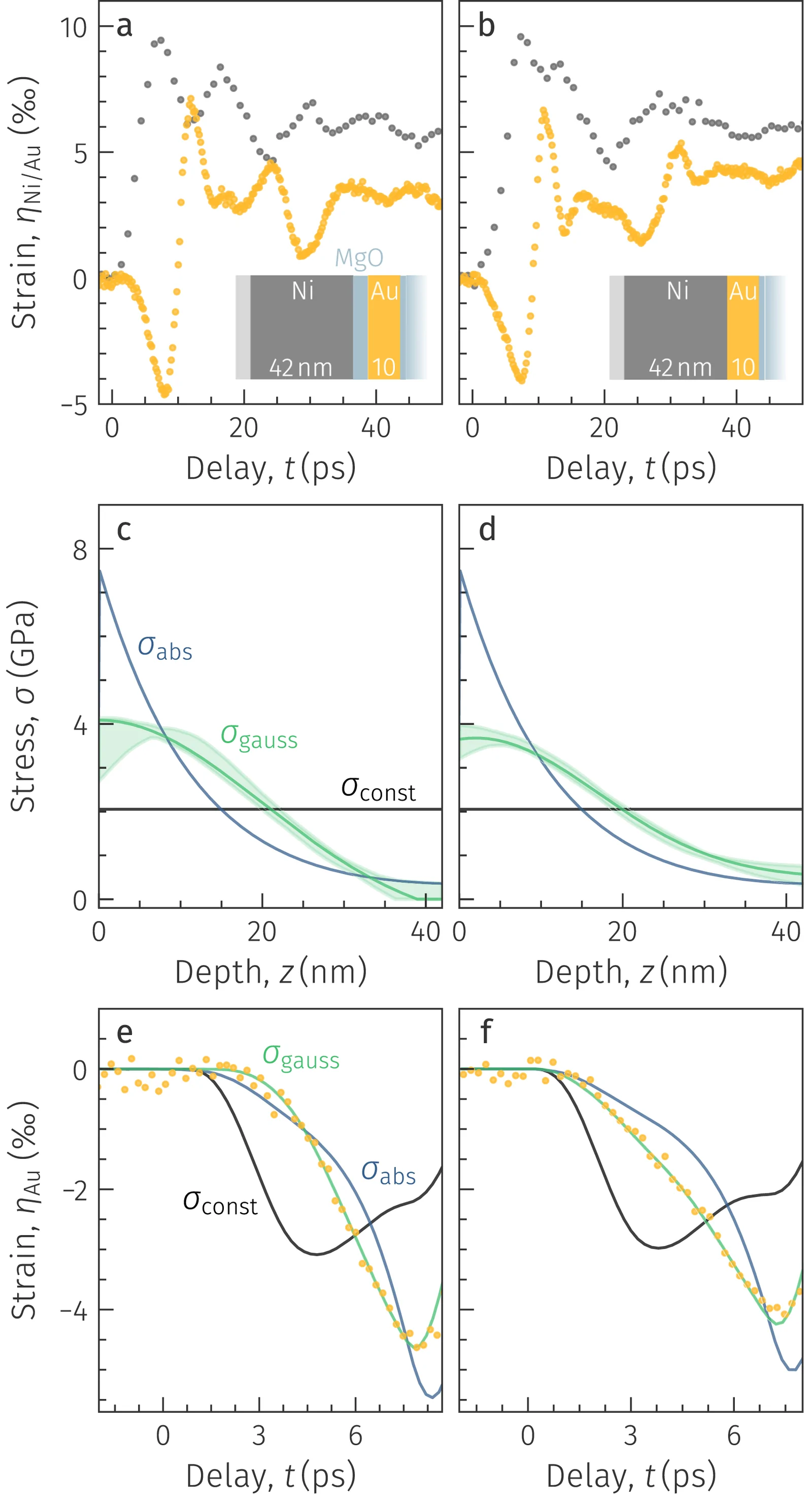
**Enhanced sensitivity to spatial stress profile for thin detection layers:** (a,b) Transient strain response of the photo-excited Ni (gray) and the buried Au (yellow) detection layer with or without an insulating MgO interlayer, respectively. (c,d) Extracted spatial stress profile (green solid line) compared to absorption profile (blue solid line) and homogeneous stress profile (black solid line). (e,f) Strain response of the Au detection layers (symbols) and the modeled strain response (solid lines) for the stress profiles in (c,d). The pronounced deviations between the measured and modeled strain responses for $\sigma_{z,\text{const}}$ and $\sigma_{z,\text{abs}}$ demonstrate the higher sensitivity of the spatial stress profiling (small area of 2σ interval in c,d) when a thinner detection layer is employed.

## 4 Thinner detection layers enhance sensitivity

In the next step, we reduce the thickness of the buried Au detection layer below the spatial width of the launched bipolar strain pulse. Under these conditions, the amplitude of the maximum compression is no longer given by the integral of the stress within the photo-excited layer but also depends on its spatial profile (see Fig. 1d). This should generally enhance the sensitivity to the spatial stress profile $\sigma_{z}$ (see Fig. S1d).

Fig. 4 displays the extraction of the spatial stress profile within 42-nm-thick Ni layers using the strain response of buried 10-nm-thick Au detection layers. We again compare heterostructures with and without an insulating MgO layer between the photo-excited Ni and buried Au detection layer. The reduced thickness of the detection layer increases the amplitude of the signatures of the bipolar strain pulse in the Au strain response (yellow symbols). In addition, we observe a finite quasi-static expansion of the Au detection layer below the MgO layer (see Fig. 4a) in contrast to the samples in Fig. 2c. We attribute this to a finite direct optical absorption due to the thinner Ni layer and a faster heating of the thin Au layer via phonons. The latter also results in a faster heating of the Au detection layer for the sample without MgO interlayer (see Fig. 4b) [45].

Again, we varied $\sigma_{z,\text{gauss}}$ in Eq. (3) to minimize the quadratic deviation between the corresponding simulated strain response of the Au detection layer and the experiment. The extracted stress profiles (solid green line) in Figs. 4c and d have shapes similar to those extracted from the strain response of the 108-nm-thick detection layer (see Fig. 3a). The most prominent difference is the approximately 6 times smaller area of the 2σ confidence interval (green shaded area). This drastically enhanced sensitivity resulting from the reduced detection-layer thickness is also reflected by significantly larger differences among the modeled strain responses (c.f. Figs. 4e,f and Fig. 3e).

**Fig. 5:**
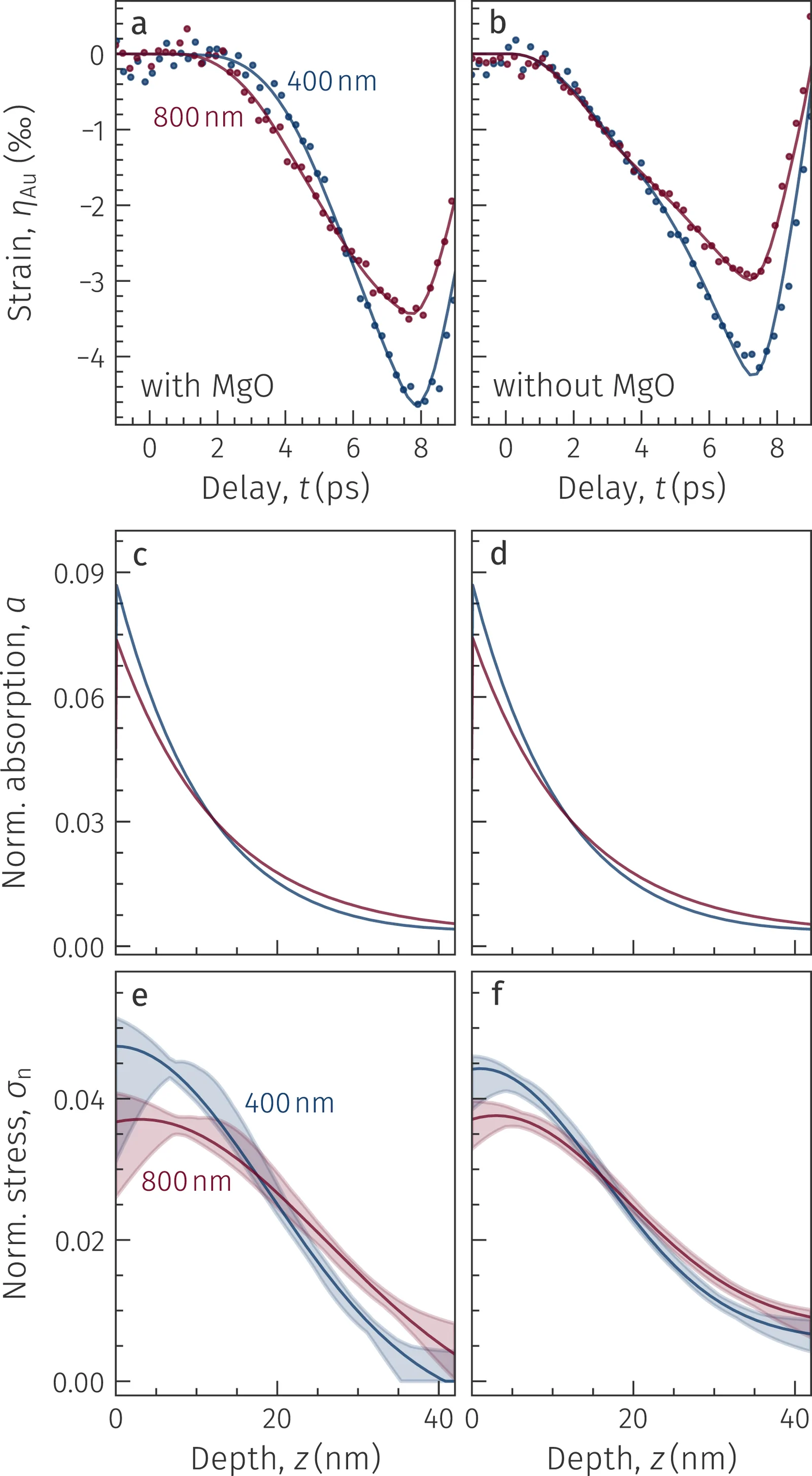
**Resolving subtle differences in the stress profile:** (a,b) Transient strain response of the Au detection layer (symbols) with or without an insulating MgO interlayer, respectively, upon excitation by 400nm (blue) and 800nm (red) pump pulses. Solid lines denote the strain response corresponding to the extracted stress profiles shown in (e,f). (c,d) Calculated normalized absorption of the 400nm (blue) and 800nm (red) pump pulses. The absorption is slightly more homogeneous for the 800nm excitation. (e,f) Extracted stress profiles (solid lines) for the 400nm (blue) and 800nm (red) excitation. Shaded areas denote the corresponding 2σ intervals. For both heterostructures, the slightly more inhomogeneous optical absorption at 400nm leads to a more inhomogeneous extracted stress profile than for 800nm excitation. The difference exceeds the 2σ intervals, indicating that the method is sensitive to subtle wavelength-dependent changes in the excitation profile.

We benchmark the potential sensitivity of spatial stress profiling via PUX by repeating the experiments in Figs. 4a and b with 800nm pump pulses instead of 400nm (see Figs. 5a,b). The two excitation conditions relate to slightly different optical absorption profiles (see Figs. 5c,d) as calculated by a multilayer transfer matrix model using the complex refractive index of Ni determined by ellipsometry on our samples. This is expected to relate to a subtle change in the spatial stress profile after the rapid electronic energy redistribution. In fact, the stresses $\sigma_{z,\text{gauss}}$ for 400 and 800nm (c.f. Figs. 5e,f) extracted from the strain response of the buried Au detection layer (see Figs. 5a,b) differ beyond the 2σ interval (shaded areas). This result for both heterostructures with and without an MgO interlayer highlights the ability of stress profiling by PUX to resolve subtle differences in the spatial profile with high precision.

## 5 Discussion

In the framework of our forward approach, the sensitivity of the stress profiling depends on the number of free parameters in the modeling and the overall agreement between the modeled and measured strain response. In this regard, three main aspects should be consider in the design of the heterostructure: (I) The signal-to-noise ratio of the transient strain of the detection layer, (II) the acoustic impedance mismatch among the layers and (III) the energy deposition to the buried detection layer. In the following, we discuss these aspects in more detail in order to derive guidelines for improving stress profiling via the sample design.

(I) The reliable determination of the Bragg peak shift in reciprocal space favors intense Bragg peaks that are well-separated in reciprocal space [20]. Thus, the detection layer should contain elements with large atomic numbers, have a lattice constant distinct from those of the other layers and be crystalline. In this regard, metal films with low requirements for sample growth techniques are advantageous. In addition, mosaic films enable reciprocal space slicing [32] a more time-efficient approach to track the shift of Bragg peaks in reciprocal space, which yields better statistics during the same measurement time. Generally, increasing the thickness of the detection layer also increases the intensity of the Bragg peak. However, at the same time the amplitude of the strain response decreases as UXRD probes the average layer strain (see Fig. 1d). The ratio of the measured strain amplitude to the square-root of the Bragg peak integral, i.e. the signal-to-noise ratio, is typically maximized for detection layer thicknesses comparable to the length scale of the stress profile. Considering the different sound velocities in Ni and Au, this corresponds approximately to the situation in Fig. 4, which means the stress profiling benefits not only from the generally higher sensitivity to the shape of the strain pulse but also from the better signal-to-noise ratio.

(II) The extraction of the spatial stress profile in the framework of our forward approach requires modeling of the propagation of the strain pulse through the heterostructure. It depends on the layer thicknesses, the sound velocities and the acoustic impedance mismatch at the interfaces. While the layer thicknesses can be independently determined by X-ray reflectivity experiments, the acoustic impedance mismatch might decisively depend on the microscopic details of the interfaces [46], [47]. Thus, it is generally advantageous to reduce the number of interfaces with an acoustic impedance mismatch within the heterostructure either by reducing the number of layers or choosing material combinations with a small acoustic impedance mismatch, i.e. similar products of density and speed of sound. Selecting only the strain response of the detection layer up to its maximum compression for the stress profile extraction (as done in this manuscript) additionally reduces the influence of the precise modeling of strain pulse reflections at acoustically mismatched interfaces.

In the case of a detection layer thinner than the spatial width of the compressive part of the strain pulse (see Fig. 4, Fig. 5), however, the shape of the initial compression is inevitably influenced by the reflection of the strain pulse at its interfaces. This means that the extracted stress profile implicitly depends on assumptions regarding the reflection of the strain pulse at this interface. Thus, selecting a detection-layer-substrate combination with minimum acoustic impedance mismatch is advantageous. In order to evaluate the resulting potential systematic errors, the measured and modeled strain responses at larger delays when the reflected strain pulse enters the detection layer again could be compared to cross-check the precise description of the interface in the model.

(III) In the case of a metallic detection layer, direct optical absorption or rapid electronic energy transport could result in a rapid excitation and concomitant expansion of the detection layer (see Fig. 2d). In this situation, the necessary description of the strain pulse launched by the detection layer itself drastically increases the number of modeling parameters and therefore the systematic error for extracting the excitation profile within the layer of interest. However, our experiments illustrate that a pronounced rapid excitation of the buried detection layer can be suppressed by insulating interlayers and even by choosing a detection layer material with a much smaller electronic heat capacity and weaker electron–phonon coupling than those of the layer of interest (see Fig. 2d). These quantities generally determine the amount of energy rapidly deposited into a layer via electrons within nanoscale metallic heterostructures [39], [42], [48]. While background-free detection of the traversing strain-pulse signature in the buried detection layer is optimal (Fig. 2c), our approach does not require the complete absence of heating in this layer. As shown in Fig. S3, slowly rising quasi-static expansion of the detection layer can be corrected with little impact on the extracted stress profile. In contrast, rapidly rising stress from direct optical absorption or ultrafast electronic energy transport can bias the extracted profile. Therefore, the reliability of the stress profiling mainly suffers when the intrinsic expansion of the detection layer evolves on a timescale comparable to that of the traversing strain pulse.

So far we have mainly discussed the influence of the spatial excitation profile on the strain response of a buried detection layer. However, the temporal evolution of the stress on the lattice given by the energy transfer among the different degrees of freedom (Eq. (2)) also influences the shape of the compression of a detection layer (see Fig. S2). The relevance of this effect crucially depends on the ratio of the timescales of stress rise and strain formation, i.e. dimension of strain pulse divided by the speed of sound. In metals the electronic stress contribution is typically comparable to that of the phonons and rises on the timescale of the laser excitation. Accordingly, the ratio of the timescales is typically close to 0 and the influence of the stress rise time on the stress profiling is negligible. This might change in case of a slow coupling between the degrees of freedom [21]. Under such conditions, the stress rise time can be independently identified by the delayed maximum compression with respect to the periodicity of the echoes of the strain pulse in the strain response of the buried detection layer at larger delays.

## 6 Conclusion

In summary, we exemplified stress profiling by PUX using buried detection layers on Ni-Au and Cu-Au heterostructures. The extracted spatial stress profiles in Ni and Cu identify the rapid redistribution of the optically deposited energy via electrons, which was found to depend strongly on the electronic thermal conductivity and the electron–phonon coupling strength. In particular, the excitation profile in the prototypical ferromagnetic Ni layer remains more similar to the absorption profile which has implications for the contributions of local and non-local demagnetization processes. Thus, Ni and Cu serve here as benchmark systems that validate the sensitivity of the method and illustrate how material-dependent electron transport shapes the initial stress profile.

Our results identify the rapid excitation of the buried detection layer as the most relevant constraint for stress profiling by PUX using buried detection layers. It can be overcome in metallic heterostructures by insulating interlayers or even detection layers with smaller electronic heat capacity and weaker electron–phonon coupling than those of the functional layer of interest. We demonstrated an enhanced sensitivity of the stress profiling by employing detection layers thinner than the spatial width of the strain pulse. Under such conditions, we resolved the influence of the slightly different absorption profile of the 400 and 800nm pump pulses on the stress profile in Ni. This highlights the high sensitivity of the quantitative experimental approach that spatially separates the generation of the strain pulse and its detection. The central experimental requirement is a crystalline buried detection layer with a distinguishable Bragg peak. Our approach widens the scope of PUX for quantitative studies of spatial energy distributions in amorphous transducer layers that are otherwise inaccessible to UXRD techniques. The present work establishes the method in well-controlled metallic heterostructures, where the extracted stress profiles can be interpreted in terms of electron-mediated energy redistribution. These benchmark experiments provide the basis for applying the approach to more complex functional materials. In future experiments, buried crystalline detection layers could be used to profile stress contributions from laser-induced phase transitions or laser-manipulated magnetic and ferroelectric order. In such systems, the extracted lattice stress profile would provide depth-resolved information on the spatial evolution of the non-equilibrium response.

## 7 Methods

### 7.1 Sample growth and characterization

The Ni-Au and Cu-Au heterostructures were grown via molecular beam epitaxy onto an MgO(001) substrate. In order to avoid oxidation of the Ni layer the metallic heterostructures were capped by a 7.5nm-thick AlO${}_{x}$ layer. We determined the thicknesses of the layers within the different sample structures via X-ray reflectivity measurements at the XPP@KMC-3 endstation at the large-scale facility BESSY II operated by the Helmholtz Gemeinschaft. In addition, we determined the complex refractive index by analyzing ellipsometry spectra using the SpectraRay 3 software package. The measurements were performed with a commercial Sentech SE850E spectroscopic ellipsometer. We obtain refractive indices of 1.70+2.56i and 2.54+4.71i for Ni and 1.21+2.10i and 0.26+4.81i for Cu at 400 and 800nm, respectively.

### 7.2 UXRD experiment

The pump-probe UXRD experiment was performed at the FemtoMAX beamline at the MAX IV Laboratory [40]. The samples were excited by 50fs-long, p-polarized laser pulses at either 400nm or 800nm, incident at approximately 20° with respect to the sample surface. For Ni the incident fluence was $19\;\text{mJ}/{\text{cm}}^{2}$ for both 400nm and 800nm. For Cu we selected a smaller incident fluence of $4\;\text{mJ}/{\text{cm}}^{2}$ due to a considerably lower damage threshold. The induced picosecond strain response was probed by tracking the position of the layer-specific Bragg peaks $Q_{z}\left(t\right)$ along the out-of-plane reciprocal coordinate $q_{z}$. The relative change of the position with respect to the value before excitation $Q_{z,0}$ defines the strain $\eta =-\frac{Q_{z}\left(t\right)-Q_{z,0}}{Q_{z}\left(t\right)}$. The transient Bragg peak position was probed by 100fs-long hard-X-ray pulses with a photon energy of 8keV and a bandwidth of approximately 2%. The X-ray pulses delivered at 10Hz were incident under 20° or 22° with respect to the sample surface for Au and Ni or Cu, respectively. The scattered X-rays were recorded by a position-sensitive area Pilatus detector from Dectris at a sample-detector distance of 50cm [40]. We followed the Bragg peak shift under fixed diffraction angles via reciprocal space slicing (RSS) [32]. Reciprocal space maps were recorded under the same conditions to determine the RSS correction factor [32] that relates the Bragg peak shift on the detector to the Bragg peak shift in reciprocal space.

### 7.3 Extracting stress profiles

We employ the modular python toolbox udkm1Dsim [41] to model the transient layer-specific average strain. The absorption profiles in Fig. 3, Fig. 4, Fig. 5 are calculated by a transfer matrix algorithm employing the experimentally determined complex refractive index of Ni and Cu and literature values for the refractive index of the other materials [38]. The densities and sound velocities of the layers are the only parameters entering the strain modeling and are taken from the literature [46] and previous works [33], [38].

First, we implement a spatio-temporal stress according to $\sigma \left(z,t\right)=\sigma_{z}\left(z\right)\cdot \sigma_{t}\left(t\right)$ considering Eqs. (2)–(5). The corresponding driven spatio-temporal strain is the solution of the elastic one-dimensional wave equation that we solve in the framework of a one-dimensional chain of masses and springs. Finally, the spatio-temporal strain response translates into a shift of the layer-specific Bragg peaks via dynamical X-ray scattering that defines the layer-specific strain response as in the experiment. In order to extract the spatial stress profile within our forward approach, we perform this modeling for an ensemble of a, b and c values and calculate the quadratic deviation $\chi^{2}$ between the modeled and experimental responses according to Eq. (1). Note, we selected the same step size of these parameters for all samples and the parameter ranges were chosen such that the entire 2σ interval was covered. The minimum $\chi^{2}$ between the modeled and measured strain responses identifies the stress profile in the experiment. As discussed in Section 5, we selected only the strain response of the Au detection layer up to its maximum compression for the comparison of the measured and modeled strain responses to reduce the influence of reflections of the strain pulse from acoustically mismatched interfaces. The 2σ interval is given by the ensemble of stress profiles $\sigma_{z,\text{gauss}}\left(a,b,c\right)$ for which the $\chi^{2}$ of the corresponding picosecond strain response is below a threshold value with respect to the minimum quadratic deviation. This threshold value is determined by the statistical F-test [49] and depends on the number of recorded delays. With an increasing number of delays this threshold value decreases which effectively increases the sensitivity of the stress profiling.

## CRediT authorship contribution statement

**M. Mattern:** Conceptualization, Formal analysis, Investigation, Methodology, Visualization, Writing – original draft, Writing – review & editing. **F.-C. Weber:** Formal analysis, Methodology, Writing – review & editing. **M. Rössle:** Formal analysis, Methodology, Writing – review & editing. **J.-E. Pudell:** Methodology, Writing – review & editing, Conceptualization. **A. Jurgilaitis:** Data curation, Methodology, Resources, Writing – review & editing. **B. Ahn:** Methodology, Writing – review & editing. **F. Baltrusch:** Methodology, Writing – review & editing. **J.C. Ekström:** Methodology, Writing – review & editing. **M. Herzog:** Methodology, Writing – review & editing, Conceptualization. **J. Jarecki:** Methodology, Writing – review & editing. **D. Kroon:** Methodology, Writing – review & editing. **L. Mehner:** Methodology, Writing – review & editing. **C. Walz:** Methodology, Writing – review & editing. **S.P. Zeuschner:** Methodology, Writing – review & editing. **J. Larsson:** Funding acquisition, Methodology, Resources, Writing – review & editing. **M. Kronseder:** Funding acquisition, Resources, Writing – review & editing. **M. Bargheer:** Conceptualization, Funding acquisition, Methodology, Writing – review & editing. **A. von Reppert:** Conceptualization, Formal analysis, Methodology, Writing – review & editing. **D. Schick:** Funding acquisition, Writing – original draft, Writing – review & editing.

## Code availability

The codes used to evaluate the data and simulate the dynamics are publicly available at https://doi.org/10.5281/zenodo.21996063.

## Declaration of competing interest

The authors declare that they have no known competing financial interests or personal relationships that could have appeared to influence the work reported in this paper.

## Data Availability

Data recorded during the experiment at the MAX IV Laboratory are publicly available at https://doi.org/10.5281/zenodo.21996063.

## Appendix A Supplementary data

MMC S1
