# Supplementary material for: Spatial stress profiling by picosecond ultrasonics with X-rays using buried detection layers
Source: Photoacoustics. 2026 Aug 17;51:100871. doi: 10.1016/j.pacs.2026.100871 (PMC13546831; doi:10.1016/j.pacs.2026.100871)
Supplement: Supplementary file 1 [file mmc1.pdf]

Supplementary material to: Spatial stress profiling by picosecond ultrasonics with X-rays using buried detection layers

M. Mattern,[1,*] F.-C. Weber,[2,3] M. Rössle,[3] J.-E. Pudell,[4] A. Jurgilaitis,[5] B. Ahn,[5] F. Baltrusch,[2] J. C. Ekström,[5] M. Herzog,[2] J. Jarecki,[1] D. Kroon,[5] L. Mehner,[2] C. Walz,[2] S. P. Zeuschner,[2] J. Larsson,[5,6,7] M. Kronseder,[8] M. Bargheer,[2,3] A. von Reppert,[2] and D. Schick[1]

[1]*Max-Born-Institute for Nonlinear Optics and Short Pulse spectroscopy, 12489 Berlin, Germany*
[2]*Institut für Physik und Astronomie, Universität Potsdam, 14476 Potsdam, Germany*
[3]*Helmholtz-Zentrum Berlin für Materialien und Energie GmbH, Wilhelm-Conrad-Röntgen Campus, BESSY II, 12489 Berlin, Germany*
[4]*European X-ray Free-Electron Laser Facility, 22869 Schenefeld, Germany*
[5]*MAX IV Laboratory, Lund University, PO Box 118, 221 00 Lund, Sweden*
[6]*Department of Physics, Lund University, PO Box 118, Lund 221 00, Sweden*
[7]*LINXS Institute of advanced Neutron and X-ray Science, Mesongatan 4, 224 84 Lund, Sweden*
[8]*Institute of Experimental and Applied Physics, University of Regensburg, 93053 Regensburg, Germany*

(Dated: June 26, 2026)

* mattern@mbi-berlin.de

S1. SENSITIVITY OF THE STRAIN RESPONSE OF PHOTO-EXCITED AND BURIED DETECTION LAYERS ON THE SPATIAL STRESS PROFILE

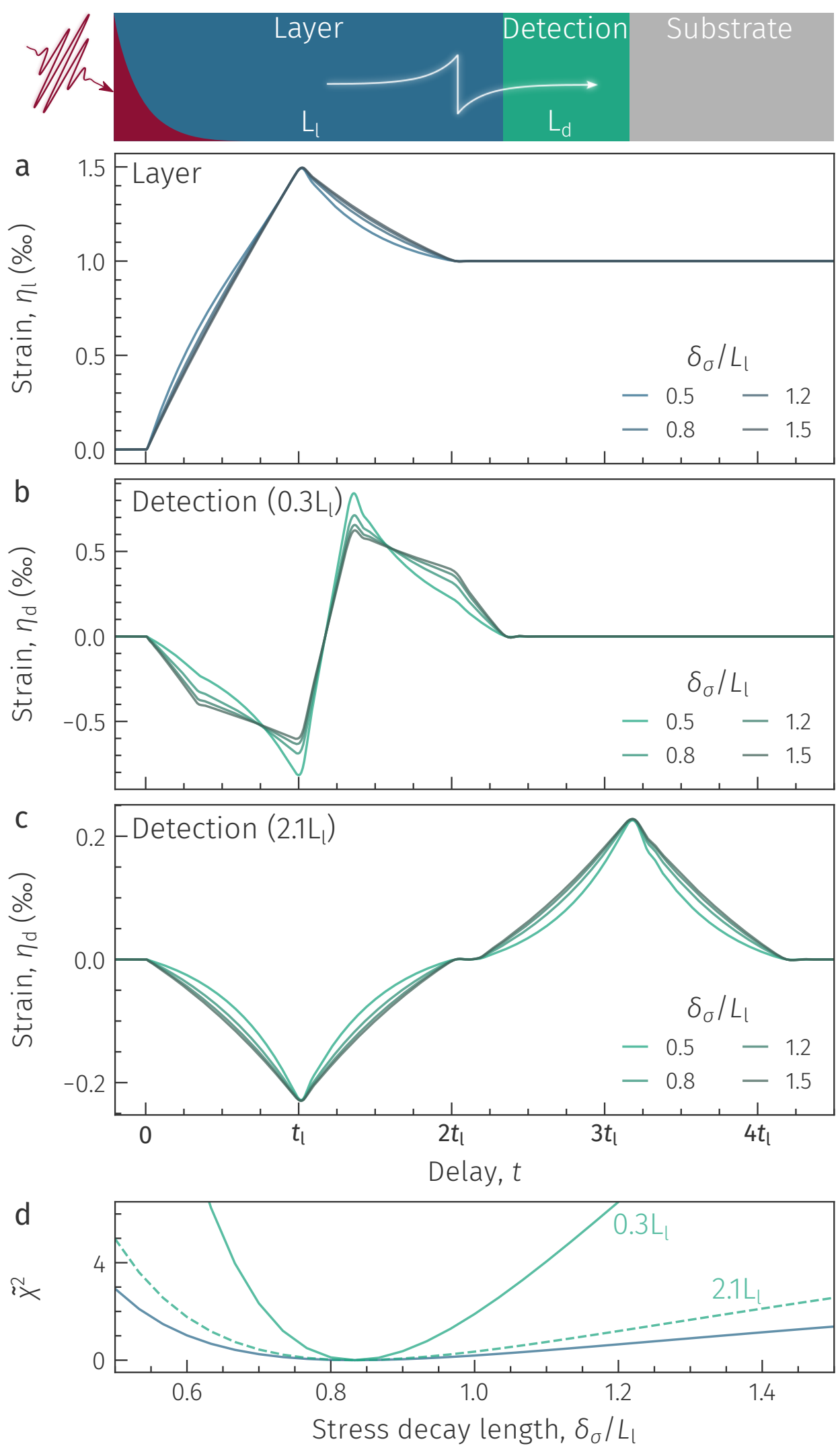

FIG. S1. **Buried detection layers enhance sensitivity to the spatial stress profile:** In a generalized heterostructure (sketch at the top) consisting of a photo-excited layer under investigation of thickness L_l and a buried detection layer of thickness L_d the laser excitation (red shaded area) launches a strain pulse (white solid line) propagating through the acoustically impedance-matched heterostructure. The stress is assumed to rise quasi-instantaneously and its spatial profile is described by $\sigma(z) = \sigma_0 \cdot e^{-z/\delta_\sigma}$ with the stress amplitude σ_0 and the stress decay length δ_σ. **a** Transient strain response of the functional layer as function of the stress decay length δ_σ relative to the thickness of the photo-excited layer L_l. The latter determines the propagation time through the layer $t_\mathrm{l} = L_\mathrm{l}/v_s$ at the speed of sound v_s. **b** The same for a buried detection layer much thinner than the dimensions of the compressive part of the strain pulse ($L_\mathrm{d} = 0.3L_\mathrm{l}$). **c** The same for a buried detection layer thicker than the dimension of the entire strain pulse ($L_\mathrm{d} = 2.1L_\mathrm{l}$). **d** The global residual $\tilde{\chi}^2$ between the modeled strain response for different δ_σ and the strain response in case of $\delta_\sigma = 0.83L_\mathrm{t}$ normalized to the maximum strain amplitude for the transducer (blue) and the buried detection layers (green) either thinner (solid line) or thicker than the photo-excited layer (dashed line). The significantly larger curvature for the detection layer highlights the larger sensitivity of the strain response of the detection layer on the spatial shape of the laser-induced stress via the launched strain pulse. This general modeling shows that the strain response of a buried detection layer is generally more sensitive on the spatial stress profile irrespective of its thickness. The 5 times higher sensitivity of the thinner detection layer compared to the thick one, mainly originates from the dependence of the maximum compression on δ_σ.

S2. INFLUENCE OF A FINITE STRESS RISE TIME ON THE LAYER-SPECIFIC STRAIN RESPONSE

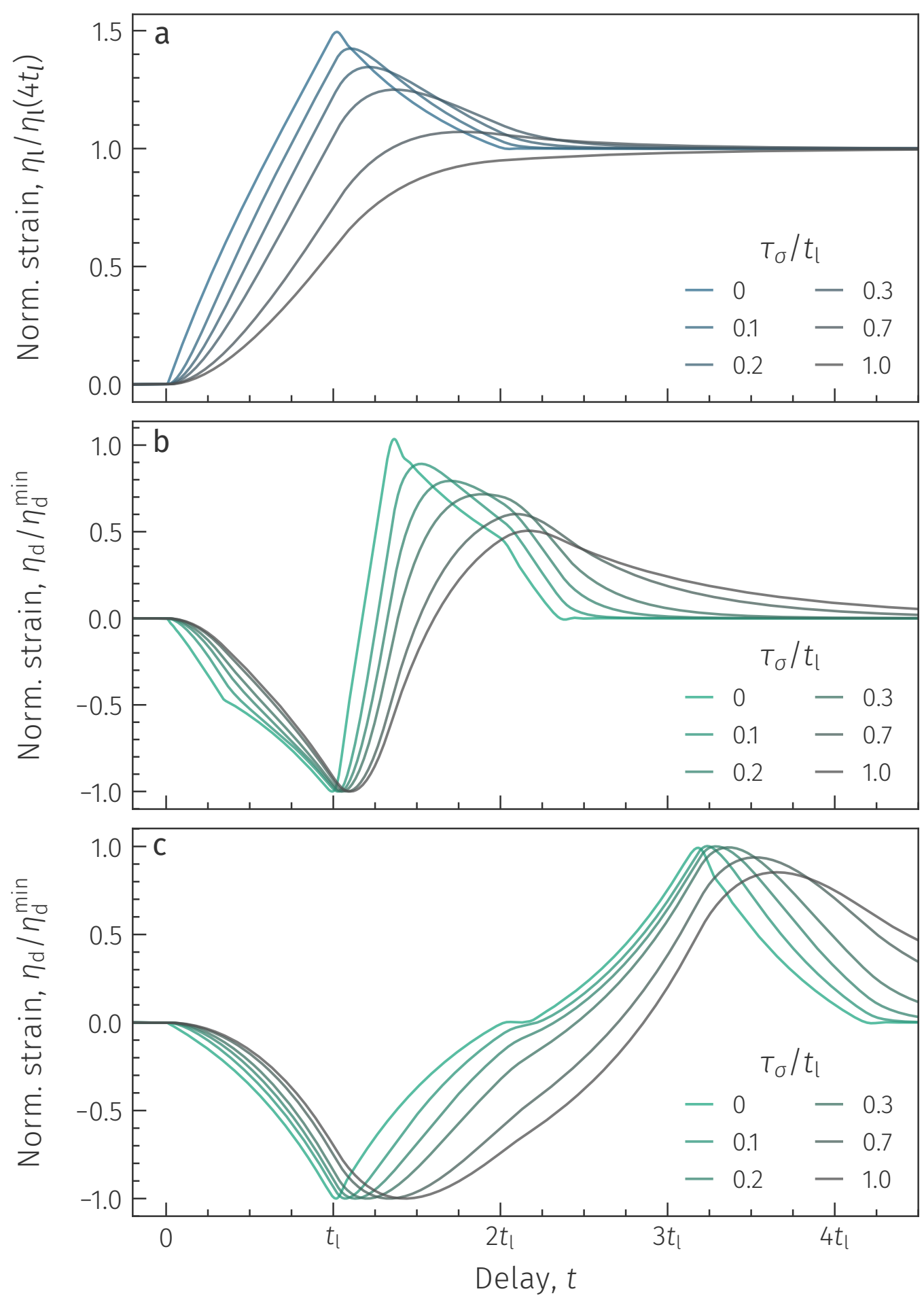

FIG. S2. **The stress-rise-time significantly affects the transient strain response only when comparable to the duration of the bipolar strain pulse: a** Strain response of the photo-excited layer in the same heterostructure as in Fig. S1 for $\delta_\sigma = L_l$ as function of the stress rise time τ_σ relative to the time it takes to propagate the strain pulse through the photo-excited layer t_l. The time-dependent stress is given by $\sigma(t) = \sigma_0 \cdot (1 - e^{-t/\tau_\sigma})$ with the stress amplitude σ_0. **b** The same for the detection layer thinner than the dimension of the compressive part of the strain pulse ($L_d = 0.3L_l$) normalized to the maximum compression. **c** The same for a buried detection layer thicker than the dimension of the entire strain pulse ($L_d = 2.1L_l$) normalized to the maximum compression. Generally, a finite stress rise time delays and broadens the signatures of the strain pulse in the layer-specific strain response. In addition, the amplitude of the strain pulse decreases as visible by the decreasing maximum strain in **a**. For the detection layer, the finite stress rise time mainly affects the shape of the strain response after the maximum compression. For $\tau_\sigma < 0.1t_l$, the influence on the initial compression of the detection layer is small, supporting the quasi-instantaneous stress approximation used for the metallic samples.

S3. CORRECTING THE INFLUENCE OF THE QUASI-STATIC EXPANSION OF THE DETECTION LAYER ON THE EXTRACTED STRESS PROFILE

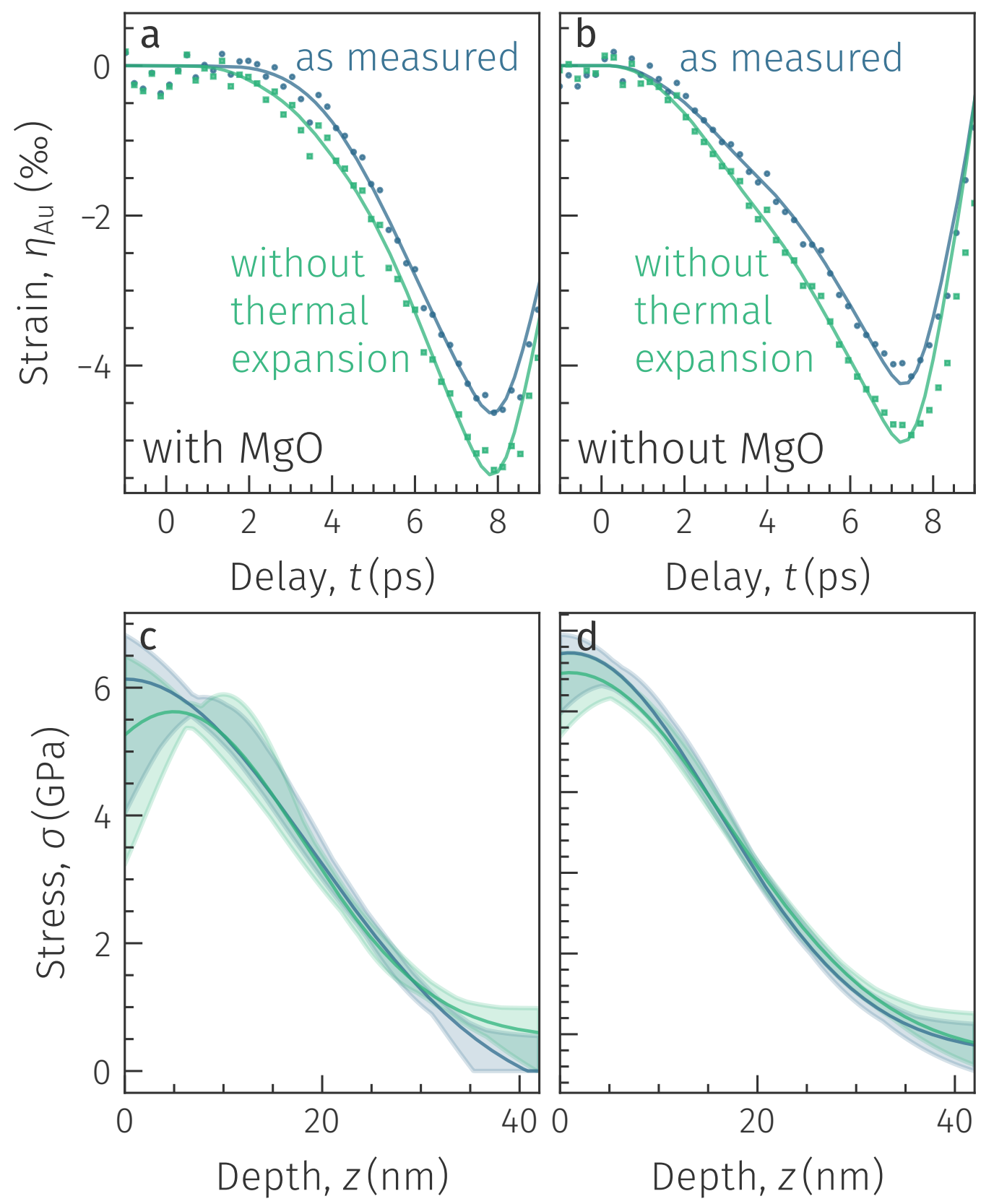

FIG. S3. **Slowly rising quasi-static expansion of the detection layer has a negligible effect on the stress profiling:** **a** Strain response (symbols) of the 10 nm-thick Au detection layer within the Ni-Au heterostructure with MgO interlayer for 400 nm excitation (see Fig. 4b) as measured (blue) and corrected for the quasi-static expansion of the Au layer (green). The solid lines denote the strain response corresponding to the best fitting Gaussian stress description (solid lines in **b**). We calculated the direct absorption in Au via a transfer matrix algorithm and calculated the influence of this direct absorption on the layer-specifc strain response. This strain response of the Au layer is subtracted from the measured data (blue symbols). This difference (green symbols) corresponds to the corrected estimate of the strain pulse signature. **b** The same for the Au detection layer within the heterostructure without MgO interlayer (see Fig. 4a) but we subtract a slowly rising expansion of Au that originates from diffusive energy transport. Due to the direct contact with Ni, the optically absorbed energy is rapidly transferred to Ni via hot electrons which suppresses a pronounced expansion of Au directly after photo-excitation. Instead we describe the rise of η_{Au} until 50 ps by a single exponential rise and subtract the best fit with the experiment from the measured strain response. **c** Extracted stress profile (solid line) in the framework of Eq. (3) and the 2σ confidence interval (colored area) employing the measured strain response (blue) and the for the quasi-static expansion of Au corrected strain response (green) for the sample with MgO interlayer. **d** The same for the heterostructure without MgO interlayer. While the slowly rising expansion of Au due to diffusive energy transport barely influences the extracted stress profile, the rapid expansion due to a photo-excited stress in Au significantly influences the extracted stress profile, especially near the Ni-MgO-interface.
